# *In situ* probe and inhibitory RNA synthesis using streamlined gene cloning with Gibson assembly

**DOI:** 10.1016/j.xpro.2022.101458

**Published:** 2022-06-14

**Authors:** Andrew Wolff, Cynthia Wagner, Julia Wolf, Daniel Lobo

**Affiliations:** 1 University of Maryland, Baltimore County, Baltimore, MD 21250, USA

**Keywords:** Developmental biology, Model Organisms, Molecular Biology

## Abstract

The synthesis of single-stranded riboprobes or double-stranded RNAs for *in situ* hybridization and gene knockdowns often use vectors that require time-consuming plasmid restriction digests and inefficient gel purifications. Here, we present a faster protocol for the simultaneous plasmid restriction digestion and Gibson assembly of vectors for the synthesis of both riboprobes and double-stranded RNAs for *in situ* and RNA interference experiments, respectively. We illustrate the protocol with planaria *in situ* and RNAi assays, but it is applicable to any organism.

## Highlights

• Simultaneous digestion and assembly of stable vectors with Gibson assembly
• Synthesis of sense and antisense riboprobes and inhibitory RNA from same plasmid
• Protocol illustrated with planaria but applicable to any organism

## Abstract (editor highlights)

Publisher’s note: Undertaking any experimental protocol requires adherence to local institutional guidelines for laboratory safety and ethics.

## Graphical abstract

**Figure:**
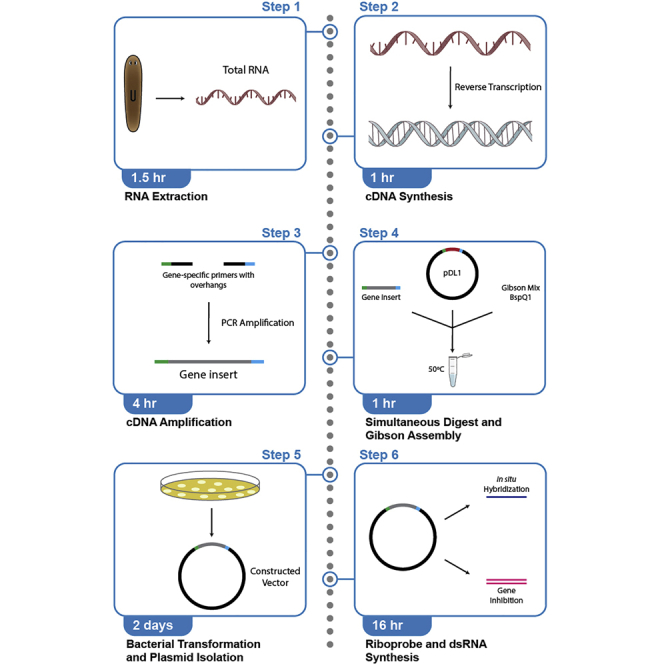

## Before you begin

The protocol below describes the specific steps for creation of vectors for synthesizing *in situ* hybridization riboprobes and double-stranded RNA (dsRNA) for RNA interference (RNAi) for the planarian flatworm *Schmidtea mediterranea*. However, this protocol can be applied for creating vectors for any organism. Negative and positive controls should be performed for both *in situ* hybridization riboprobes and dsRNAs. A negative control for riboprobes is synthesizing a probe in the sense direction. A negative control for RNAi can be the sequence for a gene of an unrelated species; we synthesize a dsRNA using the sequence for GFP, which is not native to planarians. We suggest synthesizing a vector containing a sequence like this to serve as a negative control, using a sequence not normally found in your organism. Positive controls for riboprobes and RNAi can be a sequence that has been shown previously to produce a successful *in situ* hybridization pattern or gene knockdown phenotype.

### Design primers with Gibson overhangs

**Timing: 0.5–1 h**

1. Design PCR primers specific for the gene of interest.
  a. Input in Primer3Plus (Untergasser et al., 2007), or any other primer design tool of your choice, the transcript sequence from the gene of interest to design forward and reverse primers.
    i. The length of the PCR product (i.e., the length of the gene insert) is set between 800 and 1000 bp.
    ii. Each primer is set between 20 and 24 bp.
    iii. GC content is set between 40 and 60%.
    iv. CG Clamp is set to 1.

***Note:*** When choosing primers, take a few factors into account. First, we typically check for self-dimer and hetero-dimer scores using the OligoAnalyzer tool from IDT (https://www.idtdna.com/calc/analyzer). As recommended by IDT, the ΔG value for these scores should be weaker (more positive) than -9.0 kcal/mole. Second, be sure to BLAST your primers against the transcriptome to ensure that they only produce hits for your gene of interest. Lastly, knowing the open reading frame of the transcript will also ensure a good match between the riboprobe/inhibitory RNA and the mRNA of the gene.

2. Extend the 5′ end of each primer with the appropriate overhang sequence (specific for the pDL1 plasmid) for Gibson assembly:
  a. Forward primer extended with 5′-TTAACCCTCACTAAAGGGAG-3′.
  b. Reverse primer extended with 5′-GGGATTTAGGTGACACTATAGAA-3′.
3. Synthesize primers (we typically use IDT — Integrated DNA Technologies, Inc.).

### Prepare Gibson Reaction Buffer and master mix

**Timing: 0.5–1 h**

***Note:*** This recipe is adapted from (Gibson et al., 2009), changing the Gibson Master Mix to 2× instead of 1.5×. We make and use our own to be more economical. Commercial Gibson master mixes are available and can be used in this protocol.

4. Prepare 5× Gibson Reaction Buffer.
  a. Add to 15 mL sterile falcon tube:
    i. 3 mL 1 M Tris pH 7.5.
    ii. 150 μL 2 M magnesium chloride.
    iii. 60 μL each 100 mM dNTP set.
    iv. 300 μL 1 M DTT.
    v. 1.5 g PEG 8000.
    vi. 300 μL 100 mM β-NAD.
  b. Vortex to mix.
  c. Add sterile water to 6 mL with p1000 pipettor (about 1 mL).
  d. Vortex to mix.
  e. Store in 18 aliquots of 320 μL each in 1.5 mL tubes at −20°C. The aliquots remain stable for at least 1 year at −20°C.
5. Prepare 2× Gibson Master Mix.
  a. Keep all tubes on ice or in −15°C mini-cooler at all times.
  b. Add to 1.5 mL tube with 320 μL of 5× Gibson Reaction Buffer:
    i. 0.64 μL T5 Exonuclease.
    ii. 20 μL Phusion High Fidelity Polymerase.
    iii. 160 μL Taq DNA Ligase.
    iv. 300 μL sterile water.
  c. Pipet up and down to mix.
  d. Store in 80 aliquots of 10 μL each in 0.2 mL PCR tubes at −20°C (keep them on ice during aliquoting). The aliquots remain stable for at least 1 year at −20°C.

## Key resources table

**Table:** 

REAGENT or RESOURCE	SOURCE	IDENTIFIER
**Bacterial and virus strains**		

Mix & Go! Competent Cells - DH5 Alpha	Zymo Research	T3009

**Chemicals, peptides, and recombinant proteins**		

dNTP set, 100 mM solutions	Fisher Scientific	R0181
Promega Polyethylene Glycol 8000 (PEG)	Fisher Scientific	PR-V3011
β-Nicotinamide Adenine Dinucleotide (β-NAD)	Fisher Scientific	ICN16004701
DL-Dithiothreitol (DTT)	Fisher Scientific	AC327190010
Magnesium chloride	Fisher Scientific	BP9741-10X5
Tris	VWR	97061-794
Taq DNA Ligase	New England Biolabs	M0208L
T5 Exonuclease	New England Biolabs	M0663S
Phusion High-Fidelity DNA Polymerase	New England Biolabs	M0530S
Tryptone	Fisher Scientific	BP1421-100
Yeast Extract	Fisher Scientific	BP1422-500
Sodium chloride	VWR	BT215700-1KG
Ampicillin	VWR	97061-442
Agar	Fisher Scientific	AAA1075236
BspQ1 (500 units)	New England Biolabs	R0712S
TRIzol Reagent	Fisher Scientific	15-596-026
Chloroform	Fisher Scientific	C298-500
Isopropanol	Fisher Scientific	A416-1
Ethanol	Fisher Scientific	BP2818500
dNTP Set, 100 mM Solutions	Fisher Scientific	R0181
T3 RNA Polymerase (20 U/μL)	Fisher Scientific	FEREP0101
T7 RNA Polymerase (20 U/μL)	Fisher Scientific	FEREP0111
SP6 RNA Polymerase (20 U/μL)	Fisher Scientific	FEREP0131
RNase Inhibitor, Murine	New England Biolabs	M0314L
NTP Mix (10 mM)	Fisher Scientific	18-109-017
DIG RNA Labeling Mix	MilliporeSigma	11277073910
Ammonium Acetate (5 M), RNase-free	Fisher Scientific	AM9070G
DNase I Solution (1 unit/μL), RNase-free	Fisher Scientific	PI89836
Formamide, Deionized	MilliporeSigma	S4117

**Critical commercial assays**		

PureLink PCR Purification Kit	Fisher Scientific	K310001
GeneJET Plasmid Miniprep Kit	Fisher Scientific	FERK0503
RevertAID First Strand cDNA Synthesis Kit	Fisher Scientific	FERK1621
Zymo Research DNA Clean & Concentrator-5 kit	Fisher Scientific	NC9122861

**Deposited data**		

*Schmidtea mediterranea* dd_Smed_v6 transcriptome	PlanMine	https://planmine.mpibpc.mpg.de/

**Experimental models: Organisms/strains**		

*Schmidtea mediterranea* asexual strain planaria	n/a	CIW4

**Oligonucleotides**		

Slit-1.FOR primer TTAACCCTCACTAAAGG GAGGACTTGCCAGTCACCAATCATA	Integrated DNA Technologies, Inc	N/A
Slit-1.REV primer GGGATTTAGGTGACACT ATAGAATTTTCCCATGAAATGGATAAGG	Integrated DNA Technologies, Inc	N/A
T3 Promoter Primer	Integrated DNA Technologies, Inc	51-01-20-03
SP6 Promoter Primer	Integrated DNA Technologies, Inc	51-01-19-05
T7 Promoter Primer	Integrated DNA Technologies, Inc	51-01-20-01

**Recombinant DNA**		

pDL1 plasmid	Addgene	182263
pAW2-Smed-Slit-1 plasmid	Addgene	182264

**Software and algorithms**		

Primer3Plus		https://www.bioinformatics.nl/cgi-bin/primer3plus/primer3plus.cgi
IDT OligoAnalyzer Tool		https://www.idtdna.com/calc/analyzer

## Materials and equipment

**Table:** Luria Broth (LB): Mix with 500 mL of deionized water and transfer to a 1 L bottle and autoclave.

Reagent	Amount
Tryptone	5 g
Yeast extract	2.5 g
NaCl	5 g

**Table:** LB Agar: Mix with 100 mL of deionized water and transfer to a 250 mL bottle and autoclave.

Reagent	Amount
Tryptone	1 g
Yeast extract	0.5 g
NaCl	1 g
Agar	1.5 g

## Step-by-step method details

### Total RNA extraction

**Timing: 1.5 h**

Planarian total RNA will be extracted from worms pooled from different regenerative time points as well as whole worms. Be sure to cool down the centrifuge to 4°C in advance.

1. Place worms in a 2 mL tube. Remove as much residual water as possible.

**CRITICAL:** If using another organism, be sure to include stages/time points where the gene of interest is expressed to ensure that its transcript will be included in the total RNA pool after extraction. We pool together multiple stages/time points to increase this likelihood.

2. Add 500 μL TRIzol reagent per 2–3 worms and place at −80°C for at least 1 h to quick-freeze them.

**CRITICAL:** TRIzol reagent is a toxic chemical and should be handled and disposed of safely.

3. Thaw the lysate at 20°C–22°C for 30 min to 1 h, vortexing every 10 min.
4. Centrifuge the lysate at 12,000 × *g* for 5 min at 4°C. Transfer the clear supernatant to a new tube.
5. Incubate for 5 min at 20°C–22°C to permit complete dissociation of the nucleoproteins complex.
6. Add 200 μL chloroform for 1 mL of TRIzol reagent used for lysis, then securely cap the tube. Vortex for 1 min.

**CRITICAL:** Chloroform is a toxic chemical and should be handled and disposed of safely.

7. Incubate for 3 min at 20°C–22°C. Centrifuge the sample at 12,000 × *g* for 15 min at 4°C.
8. Transfer the upper aqueous phase containing the RNA to a new 2 mL tube by angling the tube at 45° and pipetting the solution out. Avoid transferring any of the interphase or organic layer into the pipette when removing the aqueous phase.
9. Add 500 μL isopropanol per 1 mL of TRIzol reagent used for lysis to the aqueous phase. Mix for 2 s and incubate for 10 min at 20°C–22°C.
10. Centrifuge for 10 min at 12,000 × *g* at 4°C. Total RNA precipitate forms a white gel-like pellet at the bottom of the tube. Discard the supernatant without disturbing the pellet.
11. Add 1 mL of 75% ethanol for 1 mL of TRIzol reagent used for lysis.
12. Vortex the sample for 2 s, then centrifuge for 5 min at 7,500 × *g* at 4°C. Discard the supernatant without disturbing the pellet.
13. Air dry the RNA pellet for 5–10 min. Do not dry for too long, as the pellet will be difficult to resuspend.
14. Resuspend the pellet in 20–50 μL nuclease-free water by pipetting up and down. The resuspension will be dark in color.
15. Incubate in a heat block set at 55°C–60°C for 10–15 min.
16. Quantify the RNA concentration using a NanoDrop spectrophotometer or Agilent’s Bioanalyzer.
17. Store the RNA at −80°C or proceed with cDNA synthesis.

***Note:*** This protocol is adapted from (Liu and Rink, 2018) and (King and Newmark, 2013) as well as the manufacturer’s instructions for the TRIzol reagent.

### cDNA synthesis

**Timing: 1 h**

RNA is converted to cDNA to use as a template to synthesize the insert for the gene of interest in the Gibson assembly.

18. Thaw all components except the Reverse Transcriptase thoroughly and centrifuge for 2 s to collect components at the bottom of the tube before using. Place components on ice.
19. Add the following components to a 0.2 mL PCR tube in the indicated order:

**Table:** 

RNA template (1 μg)	X μL
Random Hexamer Primer (100 μM)	1 μL
Nuclease-free water	X μL
**Total Volume**	**12 μL**

20. Add the following components in the indicated order:

**Table:** 

5× reaction buffer	4 μL
RiboLock RNase Inhibitor (20 U/μL)	1 μL
10 mM dNTP Mix	2 μL
RevertAid Reverse Transcriptase (200 U/μL)	1 μL
**Total Volume**	**20 μL**

21. Mix gently and centrifuge for 2 s. Incubate the tube as follows in a thermal cycler:

**Table:** 

Step 1	5 min at 25°C
Step 2	60 min at 42°C
Step 3	5 min at 70°C
Optional step	Hold at 4°C

22. The reaction product can be used directly in PCR applications or stored at −20°C for less than one week. For longer storage, −80°C is recommended.

***Note:*** This protocol is adapted the manufacturer’s instructions for the RevertAid Reverse Transcriptase kit. Alternatively, any other cDNA synthesis kit will work here.

### cDNA amplification

**Timing: 4 h**

Using gene-specific primers with Gibson overhangs, the insert for the gene of interest is cloned from cDNA.

23. Prepare the PCR master mix on ice:

**Table:** PCR master mix (Phusion DNA Polymerase)

Reagent	Amount
cDNA template	1.5 μL
Phusion Polymerase	0.3 μL
Forward Primer (10 μM)	1.3 μL
Reverse Primer (10 μM)	1.3 μL
5× Phusion HF Buffer	5 μL
10 mM dNTPs	0.5 μL
ddH_2_O	15.1 μL
**TOTAL**	**25 μL**

24. Vortex the tubes for 2 s to mix. Run the tubes in a thermal cycler with the following conditions:

**Table:** PCR cycling conditions

Steps	Temperature	Time	Cycles
Initial Denaturation	98°C	30 s	1
Denaturation	98°C	10 s	35 cycles
Annealing	X°C	20 s	
Extension	72°C	15–30 s/kb	
Final extension	72°C	10 min	1
Hold	4°C	forever

***Note:*** Annealing temperature will depend on the primers you have designed. We recommend using the calculator from the polymerase vendor or your thermal cycler’s gradient feature to find the suitable annealing temperature for your primer pair.

25. Run 1–3 μL of the PCR(s) on an agarose to confirm a successful PCR.
26. Clean the PCR(s) according to the manufacturer’s instructions for the PureLink PCR Purification Kit.
  a. Add 4 volumes of PureLink Binding Buffer HC (B3) to 1 volume of the PCR product. Mix well.
  b. Add the sample to a PureLink Spin Column.
  c. Centrifuge the column at 20°C–22°C at 10,000 × *g* for 1 min.
  d. Discard the flow through and place the spin column into the collection tube.
  e. Add 650 μL of Wash Buffer to the column.
  f. Centrifuge the column at 20°C–22°C at 10,000 × *g* for 1 min. Discard the flow through from the collection tube and place the column into the tube.
  g. Centrifuge the column at maximum speed at 20°C–22°C for 2–3 min to remove any residual Wash Buffer. Discard the collection tube.
  h. Place the spin column in a clean 1.7 mL PureLink Elution Tube provided with the kit.
  i. Add 50 μL of Elution Buffer (or sterile water) to the center of the column.
  j. Incubate the column at 20°C–22°C for 1 min.
  k. Centrifuge the column at maximum speed for 2 min.
  l. Remove and discard the column (the recovered elution volume is ∼48 μL).

***Note:*** The PureLink purification kit has a buffer (B3) that allows for the removal of PCR products smaller than 300 bp. When running the PCR products on a gel, you will likely observe small bands distinct from the correctly-sized bands produced by the appropriate PCR amplification. We have found that small products (likely small primer products) interfere with subsequent Gibson reactions, so it is important to use Buffer B3 when purifying your PCR products.

27. Quantify the concentration of the PCR product using your preferred method (i.e., Nanodrop, spectrophotometer, etc.).
28. Store the gene insert PCR at −20°C until ready to proceed with Gibson assembly.

***Alternatives:*** High-fidelity polymerases (e.g., Phusion, Q5) are recommended to ensure high accuracy in the synthesis of your gene insert for the subsequent downstream nucleic acid synthesis products (e.g., riboprobes) to be as close to the target mRNA sequence as possible. However, we have also synthesized successful *in situ* riboprobes using Taq-based polymerases.

### Gibson assembly and bacterial transformation

**Timing: 2 days**

The PCR product from the gene of interest will be inserted into the pDL1 vector. The restriction digest of the vector and the Gibson reaction to insert the PCR product is done in the same tube.

**Figure 1:**
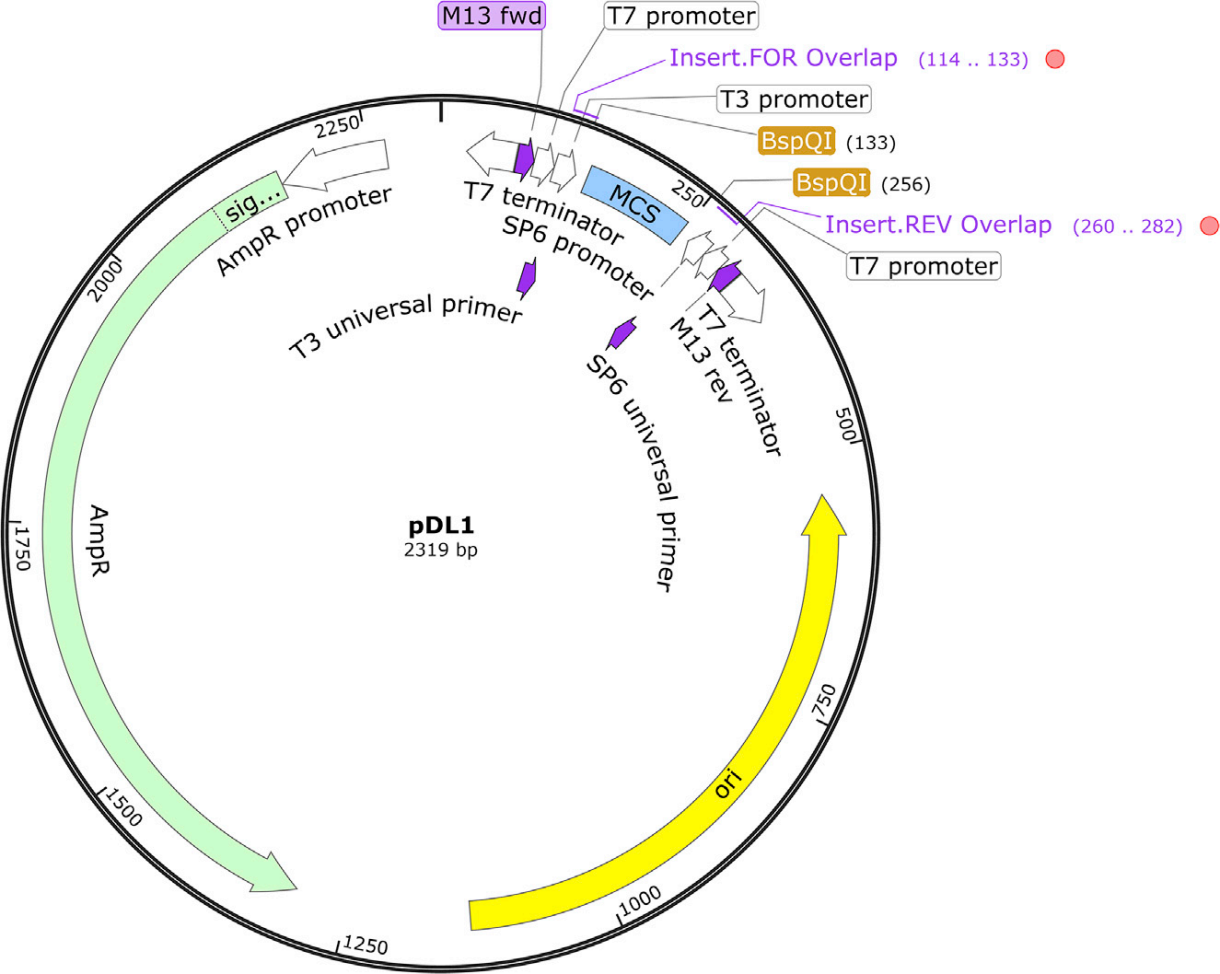
Graphical map of the pDL1 plasmid for the synthesis of single-stranded riboprobes and double-stranded RNAs The multiple cloning site (MCS) is removed through the simultaneous digest with BspQI and Gibson assembly with the gene fragment insert.

***Note:*** The pDL1 plasmid (Figure 1) can be purchased through Addgene (cat #182263). Similar to plasmid pJC53.2 for T/A Cloning (Collins et al., 2010), pDL1 includes T3 and SP6 promoters for single-stranded RNA synthesis, and two T7 promoters and terminators for dsRNA synthesis. In addition, pDL1 includes a MCS region flanked by BspQI asymmetrical restriction sites, M13 forward and reverse universal sequences before the T7 terminators for complete forward and reverse sequencing of the inserted gene, and the AmpR resistance gene. pDL1 was constructed by Gibson assembly of PCR-amplified pGEM-5Zf(+) (Promega) backbone with two synthesized gBlocks (IDT), one for each T7 terminator-M13-promoters-restriction sequence due to the presence of the two inverted T7 promoters. In the design of the gBlock sequences, one base was added between the M13 promoters and T7 terminators to prevent hairpins in the M13 promoters and reduce annealing between the two opposite T7 terminator/promoter pairs. Finally, the T3 and SP6 promoter sequences are compatible with their universal primers, as an alternative to the M13 primers for sequencing.

29. Calculate the volume needed of insert for a 3:1 molar ratio with 10–100 ng of pDL1 backbone (2196 bp after digestion of pDL1 with BspQI) as:

$$\text{insert μl }=3\cdot \text{ backbone μl}\;\cdot \;\frac{\text{backbone ng}/\text{μl}}{\text{insert ng}/\text{μl}}\;\cdot \;\frac{\text{insert bp}}{\text{backbone bp}}\;$$

We regularly use 2 μL of vector diluted at 12.5 ng/μL, resulting in:

$$\text{insert μl }=3\cdot \frac{25\text{ ng}}{\text{insert ng}/\text{μl}}\;\cdot \;\frac{\text{insert bp}}{2196\text{ bp}}$$

30. Thaw on ice two 0.2 mL PCR tubes with 10 μL 2× Gibson Master Mix (one experimental and one control).
31. Add to each PCR tube on ice:
  a. Calculated μL pDL1 backbone.
  b. Calculated μL insert DNA (experimental) OR equivalent μL nuclease-free water (control).
  c. Nuclease-free water up to 10 μL total.
  d. 0.5 μL BspQI.

***Note:*** BspQI cuts at the same site as SapI but at 50°C (the same temperature as the Gibson reaction), allowing the digest and the Gibson assembly to occur simultaneously in the same tube. Crucially, the restriction digest produces an asymmetrical cut, preventing the vector from re-linking. Thus, this results in very low background.

32. Pipet up and down to mix well.
33. Incubate at 50°C for 1 h.
34. Warm LB-Ampicillin (LB-Agar with 100 μg/mL ampicillin) plates to 37°C in plate incubator. LB-Agar plates can be stored at 4°C for up to 6 months.
35. Thaw two tubes of Mix & Go! Competent Cells on ice.

***Note:*** We found the Mix & Go! Competent Cells to be the most convenient for transformation, but other competent DH5α cells can be used.

36. Add 1 μL Gibson reaction to a tube of competent cells (both experimental and control). Incubate on ice for 5 min.
37. Plate the entire tube of competent cells onto an LB-Ampicillin plate. Incubate the plates at 37°C for 12–18 h.

### Plasmid purification and verification

**Timing: 2 days**

The completed plasmid will be grown and purified and then sequenced to verify that the insert from the gene of interest has been inserted into the backbone.

38. Prepare 15 mL LB-Ampicillin liquid media and divide equally into three 50 mL Falcon tubes. LB media can be stored at 20°C–22°C for 3–4 months.
39. Add a different single colony from the experimental LB-Ampicillin plate to each tube.
40. Incubate the tubes in a shaker at 37°C at 400 rpm for 12–18 h.

***Note:*** The shaking rotational speed indicated is for a mini-shaker. It should be adjusted according to your particular shaker’s orbital diameter for standard bacterial culture growth.

41. Using ∼3.5 mL liquid culture, miniprep the liquid cultures using the manufacturer’s instructions for the GeneJET Plasmid Miniprep Kit.
  a. Add 1.5 mL liquid culture to a 2 mL tube.
  b. Centrifuge at 10,000 × *g* for 30 s at 20°C–22°C.
  c. Remove the supernatant without disturbing the pellet.
  d. Add 2 mL liquid culture to the 2 mL tube. Centrifuge again and remove the supernatant.
  e. Add 250 μL of Resuspension Solution and vortex until the bacteria are resuspended (no cell clumps remain).
  f. Add 250 μL of Lysis Solution and invert the tube 4–6 times until the solution becomes viscous and slightly clear.
  g. Add 350 μL of Neutralization Solution and invert the tube 4–6 times. The neutralized bacterial lysate should become cloudy.
  h. Centrifuge at 12,000 × *g* for 5 min to pellet cell debris and chromosomal DNA.
  i. Transfer the supernatant to the supplied GeneJET spin column by pipetting. Avoid disturbing or transferring the white precipitate.
  j. Centrifuge at 12,000 × *g* for 1 min. Discard the flow through and place the column back into the same collection tube.
  k. Add 500 μL of Wash Solution to the GeneJET spin column. Centrifuge at 12,000 × *g* for 30–60 s and discard the flow through. Place the column back into the same collection tube.
  l. Repeat the wash procedure (previous step) using 500 μL of Wash Solution.
  m. Discard the flow through and centrifuge for an additional 1 min at 12,000 × *g* to remove residual Wash Solution. This step is essential to avoid residual ethanol in plasmid preps.
  n. Transfer the GeneJET spin column into a fresh 1.5 mL tube. Add 50 μL of Elution Buffer to the center of the spin column membrane to elute the plasmid DNA. Take care not to contact the membrane with the pipette tip. Incubate for 2 min at 20°C–22°C and centrifuge at 12,000 × *g* for 2 min.
  o. Discard the column.

***Alternatives:*** Any plasmid miniprep kit can be used here to purify the plasmids.

42. Quantify the plasmid concentrations. Purified plasmids can be stored at −20°C.
43. Run 1–3 μL of the 3 plasmids on an agarose gel along with the original pDL1 vector to observe which plasmid had a successful Gibson reaction. A successful Gibson will result in the brightest band not running as far as the brightest band of pDL1.
44. Sequence the plasmid(s) with M13 forward and reverse primers to validate the successful Gibson reaction.

***Note:*** More colonies can be grown if none of the 3 initial colonies produced a plasmid with a successful Gibson reaction. However, the Gibson reaction and digest are very efficient, so this is very unlikely.

### Synthesis of RNA probes for *in situ* hybridization

**Timing: 16 h**

The created vector is used to synthesize an RNA probe for the purpose of *in situ* hybridization.

45. Generate the DNA template for riboprobe transcription.
  a. Prepare the following PCR on ice:
    PCR master mix (Phusion DNA Polymerase)

**Table:** 

Reagent	Amount
5× Phusion HF Buffer	5 μL
10 mM dNTPs	0.5 μL
SP6 Primer (10 μM)	1.3 μL
T3 primer (10 μM)	1.3 μL
Phusion Polymerase	0.3 μL
Plasmid (100 ng)	Variable
ddH_2_O	Variable
**TOTAL**	**25 μL**

    ***Note:*** In order to have enough PCR template as input for the riboprobe transcription reaction, run multiple reactions simultaneously and pull them together before purifying the PCRs.
  b. Vortex the tubes for 2 s to mix. Run the tubes in a thermal cycler with the following conditions:
    PCR cycling conditions

**Table:** 

Steps	Temperature	Time	Cycles
Initial Denaturation	98°C	30 s	1
Denaturation	98°C	10 s	35 cycles
Annealing	58°C	30 s	
Extension	72°C	15–30 s/kb	
Final extension	72°C	10 min	1
Hold	4°C	forever
  c. Run 1–3 μL of the PCR products on an agarose gel to confirm a successful PCR.
  d. Purify the PCR products with the Zymo Research DNA Clean & Concentrator-5 kit (all centrifugation steps are between 10,000 – 16,000 × *g*):
    i. Add 5 volumes of DNA Binding Buffer to each volume of PCR product. Mix by vortexing for 2 s.
    ii. Transfer mixture to a provided Zymo-Spin Column in a Collection Tube.
    iii. Centrifuge for 30 s. Discard the flow-through.
    iv. Add 200 μL DNA Wash Buffer to the column. Centrifuge for 30 s.
    v. Repeat the wash step.
    vi. Add ≥ 6 μL DNA Elution Buffer directly to the column matrix and incubate at 20°C–22°C for one minute.
    vii. Transfer the column to a new 1.5 mL tube and centrifuge for 30 s to elute the DNA.
      ***Alternatives:*** Any PCR purification kit can be used here.
  e. Quantify the concentration of the PCR product using your preferred method (i.e., Nanodrop, spectrophotometer, etc.). Store the remainder at −20°C until ready for riboprobe transcription.
    ***Alternatives:*** As with above, high-fidelity polymerases (e.g., Phusion, Q5) are recommended to ensure high accuracy in the amplification of the PCR product that serves as the template for riboprobe synthesis.

46. Prepare the following transcription reaction mixture at 20°C–22°C in 0.2 mL PCR tubes:

**CRITICAL:** When synthesizing the riboprobe, work in an RNase-free environment and use RNase-free reagents/tubes whenever possible to prevent RNA degradation.

**Table:** Riboprobe Synthesis Reaction

Reagent	Amount
5× Transcription Buffer	5 μL
10× DIG RNA labeling mix	2.5 μL
RNase inhibitor (40 U/μL)	1.5 μL
T3 or SP6 RNA polymerase	2 μL
0.8–1 μg PCR template	Variable
ddH_2_O	Variable
**TOTAL**	**25 μL**

***Note:*** The transcription buffer is included with the enzyme (T3 or SP6). Ensure the transcription buffer is thawed and fully dissolved.

***Note:*** Using SP6 polymerase will result in the synthesis of a probe anti-sense to the gene insert sequence, while T3 polymerase will yield a probe sense to the gene insert. Both sense and anti-sense riboprobes can then be used in subsequent *in situ* hybridization experiments, with anti-sense probes used to detect the expression of the gene of interest and sense probes used as negative control.

47. Vortex the tubes for 2 s to mix and centrifuge the reaction(s).
48. Incubate the reaction(s) in a thermal cycler/water bath at 37°C for 4 h–18 h.
49. Add 2 μL DNase I to each tube. Mix well.
50. Continue to incubate the reaction(s) at 37°C for 30 min.
51. Transfer the reaction(s) to new (RNase-free) 1.5 mL tubes.
52. Add 75 μL nuclease-free water.
53. Add 80 μL 5 M ammonium acetate.
54. Add 360 μL ice-cold 100% ethanol.
55. Incubate at −20°C for 30 min to 18 h.
56. Vortex the reaction for 2 s. Centrifuge at > 12,000 × *g* for 20 min at 4°C.
57. Carefully remove the supernatant with a pipet without disturbing the pellet.
58. Wash the pellet with 500 μL ice-cold 70% ethanol.
59. Centrifuge at > 12,000 × *g* for 5 min at 4°C.
60. Carefully remove the supernatant with a pipet without disturbing the pellet.
61. Repeat the 70% ethanol wash and spin step.
62. Carefully remove the supernatant with a pipet without disturbing the pellet.
63. Air-dry the pellet for a few minutes. Do not over-dry the RNA pellet as this will make it difficult to resuspend.
64. Resuspend the pellet in 100 μL de-ionized formamide.

**CRITICAL:** Formamide is a toxic chemical and should be handled and disposed of safely.

65. Analyze 2 μL of the resulting riboprobe(s) by electrophoresis on a gel (as RNase-free as possible).
66. Store the remainder of the riboprobe(s) at −80°C.

***Alternatives:*** Here we have outlined the synthesis of a digoxigenin (DIG)-labeled riboprobe. Other labels can be used to create riboprobes, such as fluorescein and dinitrophenol (DNP).

### Synthesis of dsRNA for RNA interference

**Timing: 16 h**

The created vector is used to synthesize a double-stranded inhibitory RNA molecule for the purpose of gene knockdown.

67. Generate the DNA template for dsRNA transcription.
  a. Prepare the following PCR on ice:

**Table:** PCR master mix (Phusion DNA Polymerase)

Reagent	Amount
5× Phusion HF Buffer	10 μL
10 mM dNTPs	1 μL
T7 Primer (10 μM)	2 μL
Phusion Polymerase	0.5 μL
Plasmid (final = 4 ng/μL)	Variable
ddH_2_O	Variable
**TOTAL**	**50 μL**
  b. Vortex the tubes for 2 s. Run the tubes in a thermal cycler with the following conditions:
    PCR cycling conditions

**Table:** 

Steps	Temperature	Time	Cycles
Initial Denaturation	98°C	30 s	1
Denaturation	98°C	10 s	35 cycles
Annealing	56°C	30 s	
Extension	72°C	15–30 s/kb	
Final extension	72°C	10 min	1
Hold	4°C	forever
  c. Run 1–3 μL of the PCR products on an agarose gel to confirm a successful PCR.
  d. Purify the PCR products with the Zymo Research DNA Clean & Concentrator-5 kit (as outlined above).
    ***Alternatives:*** Any PCR purification kit can be used here.
  e. Quantify the concentration of the PCR product using your preferred method (i.e., Nanodrop, spectrophotometer, etc.).
  f. Store the remainder at −20°C until ready for riboprobe transcription.
68. Prepare the following transcription reaction mixture at 20°C–22°C in 0.2 mL PCR tubes:

**Table:** dsRNA Synthesis Reaction

Reagent	Amount
5× Transcription Buffer	10 μL
10 mM NTP mix	10 μL
RNase inhibitor (40 U/μL)	1 μL
T7 RNA polymerase	1.5 μL
1 μg PCR template	Variable
ddH_2_O	Variable
**TOTAL**	**50 μL**

***Note:*** The transcription buffer is included with the enzyme (T7). Ensure the transcription buffer is thawed and fully dissolved.

69. Vortex the tubes for 2 s to mix and centrifuge the reaction(s).
70. Incubate the reaction(s) in a thermal cycler/water bath at 37°C for 12–18 h.
71. Add 5 μL DNase I. Incubate at 20°C–22°C for 20 min.
72. Transfer the reaction(s) to new (RNase-free) 1.5 mL tubes.
73. Add 55 μL 5 M ammonium acetate.
74. Add 275 μL ice-cold 100% ethanol. Mix well.
75. Incubate at −20°C for 1 h.
76. Centrifuge at max speed for 15 min at 4°C.
77. Carefully remove the supernatant with a pipet without disturbing the pellet.
78. Wash the pellet with 700 μL ice-cold 70% ethanol.
79. Centrifuge at max speed for 15 min at 4°C.
80. Carefully remove the supernatant with a pipet without disturbing the pellet.
81. Air-dry the pellet for 5 min at 20°C–22°C. Do not over-dry the RNA pellet as this will make it difficult to resuspend.
82. Resuspend the pellet in 75 μL nuclease-free water.
83. Analyze 2 μL of the resulting dsRNA by electrophoresis on a gel. A lower percent gel such as 0.8% works well here.
84. Quantify the concentration of the dsRNA using your preferred method (i.e., Nanodrop, spectrophotometer, etc.).
85. Store the remainder at −80°C.

***Note:*** This protocol is adapted from (Rouhana et al., 2013).

## Expected outcomes

This protocol is designed to generate a plasmid vector suitable for the synthesis of antisense riboprobes for *in situ* hybridization and dsRNA for RNA interference (RNAi). Using vectors synthesized by our lab, we have successfully made riboprobes to examine in *Schmidtea mediterranea* the expression of genes using colorimetric *in situ* hybridization and synthesized dsRNA for RNAi against several genes to inhibit their expression. The use of plasmids generated using this protocol can be applied to other organisms. In general, we recommend that you utilize conventional *in situ* and RNAi methodology for your particular organism, including riboprobe length and stages for pooling RNA for cDNA synthesis. Additionally, concentrations of templates for riboprobe dsRNA synthesis can be altered to synthesize these RNA products of a concentration appropriate for your particular system.

**Figure 2:**
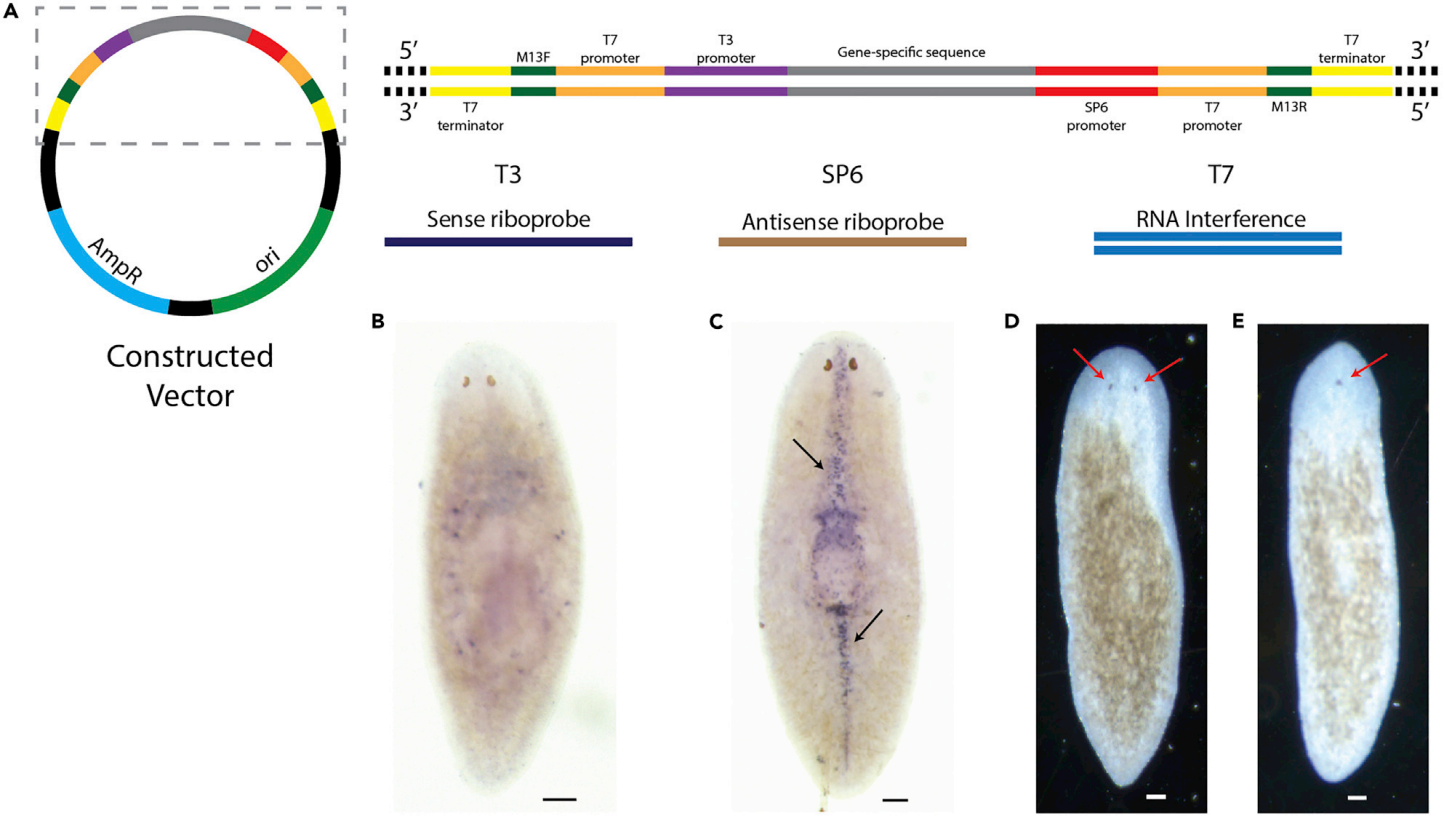
Features and use of a synthesized vector using the presented protocol for riboprobe and inhibitory RNA synthesis for planaria Smed-Slit-1 (A) The constructed vector can be used for both the synthesis of sense (with T3) and antisense (with SP6) riboprobes as well as dsRNA (with T7) for RNAi. (B) Sense riboprobes of Smed-Slit-1 produce no staining. (C) Antisense riboprobes of Smed-Slit-1 produce a characteristic midline expression pattern (arrows). (D) Head amputation in worms fed control dsRNA for GFP results in the regeneration of two eyes (red arrows). (E) Head amputation in worms fed dsRNA for Smed-Slit-1 results in the regeneration of a single eye at the midline (red arrow). Scale bars, 100 μm.

Figure 2 outlines the features of a successfully synthesized plasmid and its applications using the presented protocol. The gene-specific sequence is flanked by the promoters for T3 (sense) and SP6 (antisense) RNA polymerases. These promoters are used to synthesize sense and antisense riboprobes, respectively, containing a sequence specific for a particular gene for *in situ* hybridization. Flanking those promoters are promoter and terminator sequences for T7 polymerase, which can be used for the synthesis of double-stranded inhibitory RNA containing the gene-specific sequence. To illustrate the protocol, we synthesized a plasmid for planaria Smed-Slit-1 (pAW2-Smed-Slit-1; Addgene, 182264) and used it to generate a sense and antisense riboprobe, as well as a dsRNA to inhibit Slit-1 expression. Performing *in situ* hybridization using the synthesized riboprobes, we detected a midline expression (Figure 2C) with the antisense probe and no expression (Figure 2B) with the sense probe in whole worms. Upon delivery of dsRNA for Slit-1 and amputation of the head, we observed the collapse of the medio-lateral axis, signified by the regeneration of a single eye at the midline of the worm (Figure 2E, arrow) compared to worms fed control dsRNA that regenerate two eyes (Figure 2D, arrows. Both the expression pattern and knockdown phenotype of Slit-1 obtained are similar to previously published (Cebrià et al., 2007), validating the presented streamlined protocol.

## Limitations

A potential limitation of the outlined protocol is knowing the sequence of the gene of interest. If the sequence is not known or is poorly annotated, primers cannot be designed.

## Troubleshooting

### Problem 1

Insufficient RNA is isolated.

### Potential solution

The listed procedure is designed for isolating RNA from planarian worms. Reaction component volumes, concentrations, and incubation times may need to be adjusted for isolating RNA from other organisms to produce enough RNA for the subsequent steps.

### Problem 2

Primers with overhangs do not amplify cDNA template for PCR.

### Potential solution

The long overhangs can make PCR amplification difficult for some genes, as they are much more prone to creating self-dimers and hetero-dimers which can impede PCR. When designing the primers, ensure that they have low Delta G values in regard to self-dimers and hetero-dimers for the pair (ideal Delta G is > 7 kcal/mole). Changing the cycling conditions based on recommendations by the manufacturer of the enzyme may help as well (e.g., altering the extension time).

### Problem 3

Gibson reaction not successful.

### Potential solution

Ensure that any small primer products are removed when cleaning the PCRs. In our hands, these small products can hinder the Gibson reaction and prevent the correct insertion of the gene insert. We highly recommend using the B3 Buffer in the PureLink Purification Kit that is outlined in the protocol. To be certain the small primer products are removed, run the purified PCR on a gel as well.

### Problem 4

Sense riboprobe negative control results in high background signal.

### Potential solution

Decreasing background signal will depend on the animal that you are using, as preparation of the tissue varies from species to species. Reducing the amount of riboprobe added could reduce the background. Another cause could be that the sense probe is binding to transcripts from the opposite strand, though it is highly unlikely. BLAST the sequence of the sense probe against the transcriptome to see if any matches exist. Alternatively, probes from an unrelated species can be used as negative control.

### Problem 5

RNAi with dsRNA results in no phenotype.

### Potential solution

Increasing the dosage of dsRNA, either by concentration or number of deliveries of RNA, could produce the desired phenotype. Alternatively, you can use a different delivery method of dsRNA (e.g., injection vs. feeding).

## Resource availability

### Lead contact

Further information and requests for resources and reagents should be directed to and will be fulfilled by the lead contact, Daniel Lobo (lobo@umbc.edu).

### Materials availability

Plasmids generated in this study have been deposited to Addgene.

## Data Availability

This study did not generate or analyze any datasets or code.
